# Influence of Walker Sex and Familiarity on Scent-Marking Behavior of Juvenile and Mature Shelter Dogs

**DOI:** 10.3390/ani13233649

**Published:** 2023-11-25

**Authors:** Betty McGuire, Philippa Kok, Miles Garland, Bailey Guy, Alexandra Jackson, Scott Haber

**Affiliations:** 1 Department of Ecology and Evolutionary Biology, Cornell University, Ithaca, NY 14853, USA; msg284@cornell.edu; 2 Department of Animal Science, Cornell University, Ithaca, NY 14853, USA; pvk8@cornell.edu (P.K.); bjg224@cornell.edu (B.G.); aej34@cornell.edu (A.J.); 3 College of Arts and Sciences Communications Office, Cornell University, Ithaca, NY 14853, USA; sah67@cornell.edu

**Keywords:** urination, defecation, experience, environment, dog-human interaction

## Abstract

**Simple Summary:**

Many mammals behave differently with familiar people versus strangers, and sometimes the sex of the person is another important influence. We studied shelter dogs during walks to determine the effects on behavior of the dog’s sex and maturity and the walker’s sex and familiarity. In Study 1, unfamiliar men and unfamiliar women walked dogs. In Study 2, after walks with unfamiliar men and women, dogs were walked again when walkers were familiar. In both studies, mature males urinated at higher rates when walked by a woman than by a man, whereas mature females urinated at similar rates with women and men. Mature males and mature females were less likely to defecate when walked by a man than by a woman. Juvenile dogs were less affected than mature dogs by the walker’s sex, suggesting experience influenced responses in mature dogs. In Study 2, the effects on urination and defecation of a dog’s sex and maturity and the walker’s sex did not change over walks as dogs became familiar with walkers. Shelter dogs may be less responsive to the degree of familiarity with people than other mammals because they are directly exposed to so many people. Our results indicate that dog sex and maturity and human sex influence dog–human interactions.

**Abstract:**

Many mammals living on farms, in zoos, and in research settings behave differently with familiar people versus strangers, and the sex of the person can also influence interactions. We conducted two studies to examine the influence of a dog’s sex and maturity and a walker’s sex and familiarity on the behavior of shelter dogs during leash walks. In Study 1 with unfamiliar walkers (*n* = 113 dogs), we found that mature males urinated at higher rates when walked by a woman than by a man, whereas mature females urinated at similar rates. Mature males and mature females were less likely to defecate when walked by a man than by a woman. Juvenile dogs were generally less affected than mature dogs by a walker’s sex, suggesting a role for experience in mature dogs’ responses. In Study 2, when dogs were walked more than once by a man and a woman (*n* = 81 dogs), we found patterns of urination and defecation like those in Study 1. Importantly, the effects of the dog’s sex and maturity and the walker’s sex on dogs’ patterns of urination and defecation did not change over walks as dogs became familiar with walkers. Dogs in shelters are directly exposed to so many people that they may be less responsive to differing degrees of familiarity than mammals living in other settings. Our data indicate that dog maturity and sex and human sex influence dog–human interactions.

## 1. Introduction

Familiarity can influence interactions between humans and other mammals, including those kept on farms [1], in zoos [2], in research settings [3], and as companion animals [4]. For instance, handled piglets (*Sus scrofa*) interacted longer with their handler than with a stranger and showed less agitation and avoidance when caught by their handler [5]. Observations of several species housed at a zoo—African bush elephant, *Loxodonta africana*, Rothschild’s giraffe, *Giraffa camelopardalis rothschildi*, South American tapir, *Tapirus terrestris*, and meerkat, *Suricata suricatta*—indicated less avoidance of familiar keepers than unfamiliar keepers [6]. Laboratory rats, *Rattus norvegicus*, preferred familiar individuals to strangers when given the choice of which person to crawl up on [7] and spent more time near familiar than unfamiliar caretakers in an open field test [8]. However, preferential behavior toward familiar humans has not been found in all studies and sometimes different results are found within a species. In studies with pet cats, *Felis catus*, familiarity with humans has been shown to have either no effect [9] or a negative influence on sociability behaviors [10] and a positive influence on the duration of contact [11]. Similarly, in one study domestic horses (*Equus ferus caballus*) groomed by a familiar handler showed a lower stress response than when groomed by an unfamiliar handler [12], while in another study, horses handled by their owners and an unfamiliar handler showed similar behavioral compliance and physiological measures of stress [13].

Domestic dogs (*Canis lupus familiaris*) have been a popular model for studies of human–animal relationships. For dogs, the effects of familiarity on behavior typically have been tested using paradigms involving exposure to the owner and a stranger, although some studies have used a familiar person (not the owner) and a stranger and more rarely, all three types of people are included (owner, familiar person, and stranger). Dogs tested in the owner versus stranger paradigm exhibited more play and exploration in the presence of their owner than a stranger and following brief separations from their owner, displayed more contact-seeking behavior toward the returning owner than the stranger [14,15]. Dogs tied out in the yard of their home and approached by either their owner or a stranger gazed more, spent more time near, wagged their tails more, and barked less during the owner’s approach than the stranger’s approach [16]. When choosing to interact with either their owner or a stranger, dogs preferred their owner when tested in an unfamiliar setting but preferred the stranger in a familiar setting [4]. Although dogs preferentially attended to pointing cues given by their owners as compared to strangers [17,18], owners and strangers were equally effective as demonstrators when dogs were tested in a detour task [19]. Finally, when tested with owners, a familiar woman, and an unfamiliar woman in eight different situations, dogs always preferred their owner to the unfamiliar woman and their owner to the familiar woman during stressful situations; minor differences were found in the responses of dogs to familiar and unfamiliar women (familiar and unfamiliar men were not included [20]). 

Given the absence of owners in settings such as animal shelters and research facilities, familiarity studies conducted in these environments instead test dogs with only familiar versus unfamiliar people, and most such studies have found few or no differences in dog behavioral responses. In one study, even though shelter dogs spent more time within arm’s reach facing (but not in direct contact with) a familiar person than an unfamiliar person in the first 2 min of a 10 min test, this effect did not characterize the remaining 8 min of the test [21]. Similarly, familiarity had little or no effect on approach/withdrawal responses of shelter dogs and time spent at different distances from people who were either familiar or unfamiliar [22]. When observed during encounter tests with either a familiar caretaker or an unfamiliar person, laboratory Beagles responded in the same friendly manner to both people [23]. However, other dog breeds (Labrador retrievers, miniature schnauzers, and cocker spaniels) kept in a long-stay enriched kennel environment, where they experienced high levels of daily contact with people, preferred unfamiliar to familiar people [21]. Despite the many diverse situations and settings in which dogs have been studied, we could find no studies that examined how familiarity with a person might affect canine scent-marking behavior during leash walks. 

Several studies suggest that canine scent-marking behavior is sensitive to environmental conditions. For example, adult male dogs that used the raised-leg urinary posture characteristic of mature males temporarily switched back to the juvenile lean-forward posture, with all feet remaining on the ground, in fearful situations involving loud noises [24,25]. Consistent with this finding relating changes in urinary behavior to stressful conditions, we previously reported that under the challenging conditions of shelter life, the percent of urinations in which mature male dogs used the raised-leg posture was 73% [26] as compared to 94–97% reported for mature male dogs living in other situations [27,28,29,30]. Further, in another study, we showed that some scent-marking behaviors exhibited during leash walks by mature shelter dogs (adults and seniors) differed when the dogs were walked by an unfamiliar man versus an unfamiliar woman [31]. When walked by an unfamiliar man, male dogs were more likely to use the juvenile lean-forward posture and urinate less frequently (another characteristic of juvenile males) than when walked by an unfamiliar woman. In contrast, the sex of an unfamiliar walker did not influence urinary posture or frequency of urination in female dogs. However, both male and female dogs were less likely to defecate when walked by an unfamiliar man than by an unfamiliar woman. Previous studies of dogs in kennels, shelters, and guide dog programs also reported behavioral responses consistent with greater unease in dogs exposed to an unfamiliar man than an unfamiliar woman (e.g., less time spent near, more time spent barking at, more time with tail in the low position, more frequent lip-licking, and presence of warning behaviors such as growling and raised hackles [32,33,34,35,36,37]). Thus, based on previous scent-marking studies and other studies that assessed a wide range of different behaviors, we interpreted the behavioral differences we observed in scent-marking in our previous study [31] to reflect greater unease with unfamiliar men than unfamiliar women.

Here, we present two studies on the scent-marking behavior of shelter dogs during walks by men and women. In Study 1, we extend our previous findings [31] by including juvenile dogs along with mature dogs walked by an unfamiliar man and an unfamiliar woman. Our goal was to determine whether the behavioral responses of juveniles toward unfamiliar male and unfamiliar female walkers would differ from those of mature dogs. To our knowledge, age-related differences in how dogs respond to unfamiliar men and unfamiliar women have not been reported, and any differences found between juvenile and mature dogs could shed light on when such responses develop. In Study 2, an unfamiliar man and an unfamiliar woman walked shelter dogs, and this was followed by subsequent walks when dogs were familiar with the male and female walkers. Our goal in Study 2 was to determine whether responses of juvenile and mature dogs to unfamiliar male and unfamiliar female walkers would be maintained on subsequent walks when dogs were familiar with walkers. If uneasiness decreased over walks, this could inform shelters looking for ways to reduce fear and stress in dogs in their care.

As mentioned, we could find no reports of age-related differences in the response to an unfamiliar man as compared to an unfamiliar woman; this is because most previous studies used either mature dogs only [32,33,34,35] or mostly mature dogs (94 of 111 dogs studied) and did not examine the effects of age [36] or did not examine the interaction between the age of the dog and sex of the stranger [37]. However, there are data indicating that reactivity toward unfamiliar humans (not differentiated by sex) increases with age in dogs. In several studies, increased age was associated with an increased risk of stranger-directed aggression in pet dogs, as assessed by owner-completed questionnaires [38,39,40]. Additionally, an observational study of free-roaming village dogs in Mexico tested whether dogs of three age classes would approach a female stranger: most puppies completely approached, most juveniles partially approached, and most adults did not approach at all [41]. Given that reactivity toward unfamiliar humans seems to increase with age in dogs, in Study 1 we expected that juvenile dogs would show little or no difference in scent-marking behavior when walked by an unfamiliar man and an unfamiliar woman and that mature dogs would show responses like those found in our earlier study [31]. For Study 2, because previous studies with shelter dogs [21,22] and pet dogs [20] found little difference in their behavior when with familiar people (not the owner) and unfamiliar people, we predicted that scent-marking behavior during walks with an unfamiliar man and an unfamiliar woman would not change when dogs were familiar with their walkers, and this would characterize both juvenile and mature dogs. 

## 2. Materials and Methods

These studies were carried out under protocol 2012-0150, which was approved by Cornell University’s Institutional Animal Care and Use Committee.

### 2.1. Study Shelter

We conducted behavioral observations of dogs during walks at the Tompkins County SPCA in Ithaca NY, USA. The shelter is no-kill, open-admission with scheduled intake and uses a conversation-based approach for adoptions. Active volunteer programs exist for both cats and dogs. Dog volunteers walk, socialize, train, and sometimes groom the dogs. As previously reported, women outnumber men as dog volunteers and in most staff positions at the shelter [31]. The behavioral observations included here began in September 2018 and ran through mid-March of 2020, when the shelter closed to volunteers due to the COVID-19 pandemic (all members of our walking team volunteer at the shelter). The shelter reactivated its dog volunteer program in July 2021. We restarted our observations in September 2021 and completed them in June 2023.

### 2.2. Care and Housing of Dogs

Dogs are admitted to the rescue building where they are housed in chain link cages with an indoor space (2.2 m^2^) and an outdoor run (3.5 m^2^). They undergo a veterinary examination at intake, which includes vaccinations, a fecal exam, deworming, flea control, heartworm testing, and, in older dogs, a complete blood count/chemistry profile. A urinalysis is performed for dogs of any age if surrendering owners report urinary issues or if symptoms, such as frequent urination, are observed by shelter staff or volunteers. Dogs without a microchip receive one. Most dogs admitted to the shelter are mixed breeds; the number of purebred dogs is unknown because DNA testing is not routinely performed.

A few days after intake, dogs are behaviorally evaluated in the Pet Adoption Center, which is adjacent to the rescue building [42,43,44]. Following evaluation, they are moved to the adoption floor where they are housed in cubicles ranging in size from 5.2 to 7.3 m^2^. In both the Rescue Center and the Pet Adoption Center, dogs are almost always individually housed; exceptions include puppies from the same litter and dogs surrendered from the same household that staff judge need to be housed together; none of the dogs in our study were housed together. Each cubicle has a bed, blanket, water bowl, toys, and often a crate. Staff feed the dogs between 08:00 and 09:00 h and between 14:30 and 15:00 h. Several times a day, staff and volunteers either walk the dogs or take them to a large outdoor play yard; each day, start and end times of walks or visits to the play yard are recorded on a dry-erase board in the dog wing. Other forms of enrichment include day trips or overnight stays with volunteers and play groups of compatible dogs. All dogs wear either a buckle or martingale collar; a harness (previously fitted by staff) and leash (at least 1.8 m long) hang on a hook outside each dog’s cubicle. Most harnesses are the PetSafe^®^ Easy Walk^®^ brand (Radio Systems^®^ Corporation, Knoxville, TN, USA).

### 2.3. Data Collection

Our walking team consisted of six males (four 20–22 years of age and the remaining two, 31 and 37) and six females (five 20–22 years of age and one 64). Walks began on shelter grounds and continued across the street into a large field (16.6 ha; 42°28′20″ N, 76°26′22″ W), the substrate of which was mostly grass. A creek, forest, and other fields bordered the walking area. Each member of the team individually walked dogs at the shelter from one to three times a week. During walks, we let dogs freely investigate their surroundings and set the pace of the walk; however, they were not allowed to directly interact with other dogs or people other than their walker. There was no set walking route because adjustments had to be made during each walk, given that other volunteers with shelter dogs (and more rarely members of the public) were walking in the fields as well.

Upon arrival at the shelter, a walker checked the dry-erase board in the dog wing and chose a dog that had not been outside (either walked or in the play yard) for at least 2 h. Dog walking shifts (2 h in duration) at the shelter are scheduled for 1200, 1430, and on one day each week, there is an additional shift at 1700 h; thus, we walked the dogs about 2–3 h after their previous walk or time spent in the play yard. A walker entered the dog’s cubicle, greeted and harnessed the dog, and walked the dog out of the shelter. Behavioral observations began once outside and ended outside 20 min later, precisely timed using cell phones. During this 20 min period, we verbally recorded scent marking behaviors—each urination and defecation—using our cell phones (e.g., the voice memo app on an iPhone 12, model MN9G2LL/A, Apple Inc., Cupertino, CA, USA). We repeated these same procedures on the second walk for each dog, which had to occur at least 1 day after the first walk. If dogs remained on the adoption floor after our second walk, we walked them additional times using the same methods described for the first walks. 

We estimate that we interacted with each dog for about 30 min: (1) time spent with each dog before walks (greeting and harnessing the dog in its cubicle); (2) the 20 min of data collection during the walk; and (3) time spent after data collection had ended (walking back to the shelter, removing the harness, and again briefly interacting with the dog before leaving its cubicle). Gácsi et al. [45] found that shelter dogs form attachments to humans after 30 min of total contact (10 min over three days); these data informed our decision to categorize dogs as familiar with us after we had walked them once. We chose a 20 min observation period to be consistent with our previous study on how the sex of an unfamiliar walker influences the scent-marking behavior of dogs at this shelter [31].

After walks, we transferred the data from verbal recordings onto paper check sheets. In addition to the behavioral data collected, we photographed each dog and retrieved their demographic information from door signs and shelter records (e.g., intake date, source, identification number, and age). We used intake date to calculate time at the shelter, defined as the number of days from intake to the day of each walk with us; we also recorded days elapsed between the first walk and subsequent walks. All data were uploaded to Box, a service for data and document sharing and storage.

Over the course of data collection, we observed dogs between the ages of 4 months and 13 years. We classified dogs as juveniles (4 months to <1 year) and mature (1 year and older). To understand whether familiarity would influence dog responses to walkers, our goal was to have each dog walked at least two times by at least one male walker (when unfamiliar and familiar) and at least two times by at least one female walker (when unfamiliar and familiar); we described these dogs as having complete data for Study 2. Ideally, the second walks occurred within a few days of the first walks. However, it was not always possible in the shelter environment to collect complete data or for the second walks to occur within a few days of the first walks. Causes of incomplete data collection or longer periods between first and second walks included dogs being adopted throughout our research or transferred to other shelters or rescues; dog meets with potential adopters (and also with their resident dogs) that understandably took priority over our walks; behavioral or medical issues requiring removal of dogs from the adoption floor for a period of time, thereby lengthening the days elapsed between first and subsequent walks; and dogs temporarily removed from the shelter for day trips, overnight stays, or fosters with volunteers. Although necessary, our collecting data only on dogs that had not been out for at least 2 h also limited the dogs we could observe at the shelter on any day when other dog volunteers were present. Finally, we excluded data from two dogs with health conditions (one was obese and the other arthritic) and two dogs that were very fearful of strangers and not released for walks with volunteers until more than 2 months after their admission to the shelter (most dogs arrive on the adoption floor about 10–14 days after admission to the shelter and are immediately available for walking by volunteers). 

By the end of data collection, we had 32 dogs with incomplete data (4 juvenile and 6 mature males and 3 juvenile and 19 mature females), defined as walked once by at least one unfamiliar man and at least one unfamiliar woman but without subsequent walks by both the same man and the same woman. We had complete data for 81 dogs (9 juvenile and 30 mature males and 12 juvenile and 30 mature females). For these 81 dogs, the second walk typically occurred within a few days of the first walk but there were exceptions because of the challenges described when studying dogs in a shelter environment (median = 3 days after first walk; 67% of second walks occurred from 1 to 7 days after the first walk; 26% from 8 to 14 days after the first walk and 6% from 15 to 21 days; and three walks occurred 23, 28, and 35 days after the first walk). The three second walks with the longest times elapsed from the first walk involved dogs that were walked additional times after the second walk. Thirteen of the 81 dogs were walked twice by the same man and twice by the same woman (i.e., no additional walks). Thirty-one dogs were walked more than two times by both the same man and the same woman, and thirty-seven were walked more than two times by either the same man or the same woman but not by both. Overall, number of additional walks per dog ranged from one to thirty-two, with most dogs being walked 1–3 times after their second walk. We used first walks from dogs with incomplete data (*n* = 32) and complete data (*n* = 81) to extend our previous study [31]; thus, we had 113 dogs in Study 1. We used first and subsequent walks from the 81 dogs with complete data to examine the effects of familiarity on dog behavior in Study 2. Note that data from three mature females and six mature males used in our previous study [31] were included in Studies 1 and 2; data from their second and additional walks were not included in our previous study, which focused only on first walks when walkers were unfamiliar. These nine were the only dogs from our previous study that had some second walks and sometimes additional walks as well. Two other dogs (Bru Bru and Mega) from this same period (2018–2019) were not part of our previous study and were included here. Table S1 contains all data from Studies 1 and 2 (Table S1: Data on Urination and Defecation by Shelter Dogs).

Most dogs were spayed or neutered before arrival on the adoption floor, and all were spayed or neutered before adoption. In an earlier study of scent-marking behavior displayed by mature dogs during walks at this shelter and another local shelter, one of us (BM) found that female urination rates did not change after spaying, but male urination rates decreased after neutering [46]. Thus, we also recorded the reproductive condition of dogs at the time of each walk. Of the 113 dogs with either incomplete or complete data, 45 of the 49 males were neutered for all their walks; one juvenile male was intact for all his walks and three juvenile males were intact for their first walks and either intact or neutered for subsequent walks. Of the 64 female dogs with either incomplete or complete data, 61 were spayed for all their walks; one adult was intact for all her walks, and one juvenile and one adult were intact for some of their first walks and spayed for all other walks.

### 2.4. Statistical Analyses

In Studies 1 and 2, we summarized data into means and standard deviations for urination rate and into percentages for defecation. For all analyses, we used R version 4.3.0 [47] and the following packages: glmmTMB [48] and emmeans [49].

#### 2.4.1. Study 1

We used a linear mixed effects model to model urination rate (total number of urinations/20 min) as a function of the sex of dog, sex of walker, maturity status of dog (juvenile versus mature), and all two-way and three-way interactions of those variables, along with the additional fixed effect of weeks at shelter (we converted days to weeks); we included dog ID and walker ID as random effects. We used Cohen’s *d* to estimate effect sizes. A generalized linear mixed effects model with a binomial distribution and a logit link was used to model defecation with the same variables described for urination rate. 

#### 2.4.2. Study 2

For urination rate, we used a linear mixed effects model with the sex of dog, sex of walker, maturity status of dog, and all two-way and three-way interactions of those variables, along with additional fixed effects of weeks at shelter and walk number. Random effects of dog ID, dog ID interacted with sex of the walker, and walker ID, as well as a random slope of the walk number and weeks at the shelter for each dog were included in the model. We also examined two-way interactions between the walk number and sex of dog, sex of walker, and maturity status of dog to examine whether the effects of these variables changed over walks as dogs became increasingly familiar with walkers. We used Cohen’s *d* to estimate effect sizes.

We used a generalized linear mixed effects model with a binomial distribution and a logit link to model defecation with the same variables described for urination rate except that random effects only included dog ID, dog ID interacted with sex of the walker, and walker ID. As with urination rate, we also examined two-way interactions between walk number and the sex of dog, sex of walker, and maturity status of dog.

## 3. Results

### 3.1. Study 1: First Walks

Table 1 shows descriptive statistics for the two scent-marking behaviors in relation to the sex of dog, sex of unfamiliar walker, and maturity status of dog; this information is meant to provide a general overview of the raw data.

#### 3.1.1. Urination

**Figure 1:**
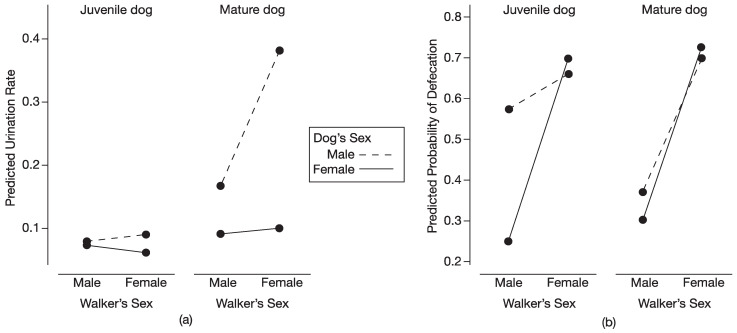
Scent-marking behaviors of dogs in relation to sex of dog, sex of unfamiliar walker, and maturity status of dog; data represent estimated marginal means from models. (**a**) Predicted rates of urination by male and female juvenile dogs and male and female mature dogs when walked by male and female walkers; (**b**) Predicted probabilities of defecation by male and female juvenile dogs and male and female mature dogs when walked by male and female walkers. Walks were 20 min in duration (*n* = 113 dogs).

We found a significant three-way interaction between the sex of the dog, the sex of the walker, and the maturity status of the dog for rate of urination (total number of urinations/20 min; *F* = 7.98, *d.f.* = 1, 107.83, *p* < 0.01). Mature male dogs walked by an unfamiliar woman had higher rates of urination than when walked by an unfamiliar man (*d* = 2.12); in contrast, mature female dogs had similar rates of urination when walked by an unfamiliar woman and by an unfamiliar man (*d* = 0.09; Figure 1a). The sex of an unfamiliar walker did not influence the rates of urination in either juvenile male (*d* = 0.05) or juvenile female dogs (*d* = 0.20; Figure 1a).

When considering sex differences in the rates of urination, mature male dogs had higher rates of urination than mature female dogs, when walked by both an unfamiliar man (*d* = 0.78) and an unfamiliar woman (*d* = 2.81; Figure 1a). For juvenile dogs, there was no sex difference in the rates of urination when walked by either an unfamiliar man (*d* = 0.09) or by an unfamiliar woman (*d* = 0.34; Figure 1a).

For the dog’s maturity status, mature male dogs had higher rates of urination than juvenile male dogs when walked by an unfamiliar man (*d* = 0.97) and an unfamiliar woman (*d* = 3.04; Figure 1a). Maturity status did not affect the rates of urination by female dogs when walked by either an unfamiliar man (*d* = 0.28) or an unfamiliar woman (*d* = 0.56; Figure 1a).

#### 3.1.2. Defecation

We found a significant effect of the sex of the walker on the likelihood that dogs would defecate during a walk (X^2^ = 7.71, *d.f.* = 1, *p* < 0.01; Figure 1b). A dog had a 0.699 probability of defecation when walked by a woman and a 0.374 probability when walked by a man. The odds that a dog would defecate with a female walker were 3.9 times higher than with a male walker (*p* < 0.01). Contrasts revealed that almost all groups–juvenile females, mature females, and mature males–had a significantly greater likelihood of defecation when walked by a woman than by a man; the juvenile males did not have a significantly greater likelihood, although their pattern was in the same direction as the other groups (Figure 1b).

### 3.2. Study 2: All Walks

#### 3.2.1. Urination

Table 2 shows descriptive statistics for the rates of urination for walks one through four, as a sample of the raw data collected for all walks. None of the two-way interactions between the sex of the dog, sex of the walker, and maturity status of the dog with walk number was significant, indicating that the effects of these variables on the rate of urination did not change over walks as dogs became more familiar with individual walkers. We dropped these two-way interactions from the final model.

**Figure 2:**
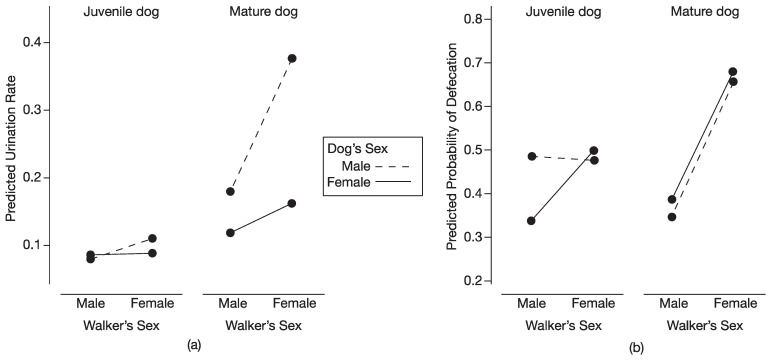
Scent-marking behaviors of dogs in relation to sex of dog, sex of walker, and maturity status of dog; data represent estimated marginal means from models. (**a**) Predicted rates of urination by male and female juvenile dogs and male and female mature dogs when walked by male and female walkers; (**b**) Predicted probabilities of defecation by male and female juvenile dogs and male and female mature dogs when walked by male and female walkers. Walks were 20 min in duration and data are from at least the first and second walk of each dog (*n* = 81 dogs); many dogs had additional walks. Given we found no significant interaction between walk number and sex of dog, sex of walker, or maturity of dog, these interactions were dropped from models; thus, the predicted values in figure are averaged across all walks of each dog.

As in Study 1, we found a significant three-way interaction between the sex of the dog, sex of the walker, and maturity status of the dog for the rate of urination (*F* = 5.31, *d.f.* = 1, 74.7, *p* < 0.05). Mature male dogs walked by a woman had higher rates of urination than when walked by a man (*d* = 3.30); in contrast, mature female dogs had similar rates of urination when walked by a woman and by a man (*d* = 0.59; Figure 2a). The sex of walker did not influence the rates of urination in either juvenile males (*d* = 0.49) or juvenile females (*d* = 0.10; Figure 2a).

When considering sex differences in rates of urination, mature male dogs walked by a woman had higher rates of urination than mature female dogs walked by a woman (*d* = 3.61); there was a trend (*p* < 0.06) for mature male dogs walked by a man to have higher rates of urination than mature female dogs walked by a man (*d* = 0.90; Figure 2a). For juvenile dogs, there was no sex difference in the rates of urination when walked by either a man (*d* = 0.19) or a woman (*d* = 0.40; Figure 2a).

Regarding the dog’s maturity status, mature male dogs walked by a man had higher rates of urination than juvenile males walked by a man (*d* = 1.49; Figure 2a). Similarly, mature male dogs walked by a woman had higher rates of urination than juvenile males walked by a woman (*d* = 4.30; Figure 2a). Maturity status did not affect rates of urination by female dogs when walked by either a man (*d* = 0.40) or by a woman (*d* = 1.09; Figure 2a).

Rate of urination decreased with weeks at shelter (*F* = 6.33, *d.f.* = 1, 15.2, *p* < 0.05) but increased with walk number (*F* = 18.24, *d.f.* = 1, 38.7, *p* < 0.001).

#### 3.2.2. Defecation

To provide a sample of the raw data collected during all walks, Table 3 shows the percentage of walks in which defecation occurred for the first four walks. None of the two-way interactions between the sex of the dog, sex of the walker, and maturity status of the dog with walk number was significant, indicating that the effects of these variables on the likelihood of defecation did not change over walks as dogs became more familiar with walkers. We dropped these two-way interactions from the final model.

For likelihood that dogs would defecate during a walk, we found a significant interaction between the sex of the walker and maturity status of the dog (X^2^ = 4.17, *d.f.* = 1, *p* < 0.05; Figure 2b). The probability of defecation by a juvenile dog was similar when walked by either a woman (0.470) or a man (0.401). In contrast, the probability of defecation by a mature dog was higher when walked by a woman (0.676) than by a man (0.363). The odds that a mature dog would defecate when walked by a woman were 3.66 times larger than when walked by a man (*p* < 0.05).

## 4. Discussion

### 4.1. Study 1: First Walks

Consistent with our previous findings at this shelter [31], mature male dogs had higher rates of urination when walked by an unfamiliar woman than by an unfamiliar man, and mature female dogs had similar rates of urination when walked by an unfamiliar woman and by an unfamiliar man. As before, we interpret the reduced rates of urination by mature male dogs to reflect greater uneasiness with unfamiliar men than with unfamiliar women. A pattern of greater unease with unfamiliar men, sometimes displayed by both male and female dogs, has been reported in several studies using diverse behavioral measures, which included less time spent near, more time spent barking at, more time with tail in the low position, more frequent lip-licking, and the presence of warning behaviors such as raising hackles and growling [32,33,34,35,36,37]. Additionally, the urinary behavior of male dogs—both posture and rate of urination—appears generally sensitive to fearful or stressful conditions [24,25,26,31].

Our inclusion of juvenile dogs in the present study revealed that the sex of an unfamiliar walker did not influence rates of urination by either juvenile males or juvenile females. Some studies on stranger-directed aggression in dogs found that avoidance and warning or aggressive responses to unfamiliar people increase with age [38,39,40,41]. Age-related increases in reactivity to unfamiliar people may reflect the cumulative effect of experiences with strangers perceived by dogs as threatening [39]. In the same way, cumulative experiences with unfamiliar men perceived as threatening might explain why we found that mature male dogs, but not juvenile male dogs, urinated at lower rates when walked by an unfamiliar man than by an unfamiliar woman. However, we did not directly test this possibility. We also found sex differences in the rates of urination for mature dogs (males had higher rates than females) but not for juveniles. Regardless of the sex of the walker, mature males had higher urination rates than juvenile males, whereas mature females and juvenile females had similar rates of urination. These findings regarding sex differences in and effects of maturation on rates of urination have been well documented for dogs [30,50,51].

Dogs were more likely to defecate when walked by an unfamiliar woman (0.699 probability) than by an unfamiliar man (0.374 probability), again suggesting greater uneasiness with unfamiliar men. The present results agree with our previous findings on the likelihood of defecation for mature dogs [31]. A closer look at our current data revealed that the observed pattern of a greater likelihood of defecation with a female walker was significant for mature males, mature females, and juvenile females but not for juvenile males, although their response was in the same direction as the other groups. 

We did not determine the specific cues used by dogs to discriminate the sex of unfamiliar walkers. Potential cues include tactile, auditory, visual, and olfactory stimuli. Subtle sex differences in petting techniques appeared responsible for lower cortisol levels found in shelter dogs petted by women than in those petted by men [52]. In subsequent studies when men and women were trained to use a standardized petting technique, reductions in cortisol levels were similar in dogs petted by men and by women [53,54]. Shih et al. [36] found that male walkers made more frequent physical contact with shelter dogs than did female walkers, who were more likely to talk to dogs during walks and use high-pitched voices. Pet dogs were better at matching human male voices to images of male faces in comparison to human female voices and faces; the authors suggested that dogs’ general wariness of men might promote the learning of male facial and vocal characteristics [55]. Finally, given the keen olfactory sense of dogs [56], it would not be surprising if they used olfactory cues to distinguish male and female walkers. Indeed, laboratory rats and mice discriminate experimenter sex using androgen-based olfactory cues and exhibit a strong physiological stress response in the presence of male, but not female, stimuli [57]. 

### 4.2. Study 2: All Walks

For both rate of urination and likelihood of defecation, none of the two-way interactions between the walk number and sex of the dog, sex of the walker, and maturity status of the dog was significant, indicating that the effects of these variables on urination and defecation did not change over walks as the dogs become familiar with walkers. Our findings agree with those of other studies reporting little difference in the response of dogs to familiar people (not the owner) and unfamiliar people [20,21,22,23] and stand in contrast to the preferential behavior typically shown by pet dogs to their owners over strangers [14,15,16,17,18]. Even more so than dogs in homes, dogs in shelters might be expected to display similar behavior in the presence of unfamiliar and familiar people because of their novel and challenging environment. While in the shelter, dogs have direct physical contact with many different people of varying degrees of familiarity (e.g., at our study shelter, veterinarians, veterinary students, shelter staff, volunteers, and members of the public), which might reduce their likelihood of behaving differently when with unfamiliar and familiar people. For comparison, most pet dogs living in a community in Cheshire England were estimated by their owners to interact with 3–5 people outside their household each week [58].

The patterns found for urination in Study 2, using data from all the walks, were very similar to those found in Study 1, using data only from the first walks. The single exception concerned sex differences in the rates of urination. In both Study 1 and Study 2, mature male dogs walked by a woman had higher rates of urination than mature female dogs walked by a woman. However, the significant finding in Study 1 that mature male dogs walked by a man had higher rates of urination than mature female dogs walked by a man was instead a nonsignificant trend in Study 2 (*p* < 0.06), perhaps reflecting smaller sample sizes of mature dogs in Study 2 (*n* = 30 males; *n* = 30 females) than in Study 1 (*n* = 36 males; *n* = 49 females). 

For likelihood of defecation during walks in Study 1, we detected a significant effect of the sex of the walker, with dogs more likely to defecate when walked by an unfamiliar woman than by an unfamiliar man. In Study 2, we found a significant interaction between the sex of the walker and maturity status of the dog. The probability of defecating by a juvenile dog was similar when walked by a woman (0.470) and by a man (0.401), whereas the probability of defecating by a mature dog was significantly higher when walked by a woman (0.676) than by a man (0.363). Indications that juveniles might behave differently than mature dogs were evident in Study 1, but only for juvenile males, who were the one group not to show a significantly higher probability of defecation when walked by a woman than by a man, although their pattern was the same as in other groups. It is unclear why juvenile females showed a significantly higher likelihood of defecation when walked by a woman than by a man in Study 1 but not in Study 2; their overall pattern, however, was similar across the two studies and sample sizes were somewhat smaller in Study 2 (*n* = 12) than in Study 1 (*n* = 15).

### 4.3. Limitations

Our studies have several limitations. First, we did not control for the age of the walker, which ranged from low-twenties to mid-sixties. Some mammals can discriminate human age as evidenced by their behavioral and physiological responses to certain stimuli. African bush elephants (*Loxodonta africana*) use auditory cues to differentiate men, who pose a significant hunting threat, from boys, who do not and show more defensive bunching and investigative sniffing after playbacks of men’s voices [59]. Domestic horses (*Equus ferus caballus*) use both auditory and visual cues to differentiate adults from children and show increased heart rates during children’s vocalizations [60]. However, Koda and Shimoju [33] found that dogs behaved in a similar way toward unfamiliar women (from 20 to 40 years old) and unfamiliar girls (from 8 to 13 years old); they did not include a comparison of unfamiliar men and unfamiliar boys in their study. These data on dogs suggest that at least in the case of human females, dogs respond in a similar way to individuals of different ages. Other limitations relate to the challenges of collecting data over time on individual dogs in shelters. Throughout our studies, dogs were adopted and sometimes returned, or temporarily unavailable to us due to in-shelter activities (e.g., meeting potential adopters and their dogs, and either medical or behavioral issues requiring removal from the adoption floor for 10 or more days) or out-of-shelter activities (e.g., short-term fosters and day trips with volunteers). Longer times than ideal between first and second walks for some dogs in Study 2 resulted from both in-shelter and out-of-shelter activities. Nevertheless, by including all walks for each dog we hoped to reduce the impact of those initial delays. Finally, whereas our sample sizes for mature dogs might be considered moderate (Study 1, 36 males and 49 females; Study 2, 30 males and 30 females), our sample sizes for juvenile dogs (Study 1, 13 males and 15 females; Study 2, 9 males and 12 females) were less than half those of mature dogs. The smaller number of juveniles likely reflects the shorter lengths of stay for juvenile dogs than mature dogs at our study shelter [44].

## 5. Conclusions

In Study 1 (first walks), we extended findings from our previous study showing that the sex of an unfamiliar person influences the scent-marking behaviors of mature shelter dogs [31], further strengthening our suggestion that researchers studying mature shelter dogs (and perhaps all mature dogs) should report the sex of all personnel involved in handling and data collection. We also extended our previous findings by including juvenile dogs, whose rate of urination was unaffected by the sex of an unfamiliar walker and the likelihood of defecation was unaffected in males but not females. The generally greater responsivity of mature dogs to the sex of an unfamiliar person may reflect cumulative experiences with male strangers perceived as threatening, although this was not tested here. 

In Study 2 (all walks), we found that the effects on urination and defecation of the dogs’ sex and maturity and the walkers’ sex did not change over walks as dogs became familiar with walkers. We suggest that shelter dogs have direct contact with so many different people of varying degrees of familiarity that they may be less responsive than other mammals–for example, those on farms, in zoos, and in research environments–to familiar versus unfamiliar people. 

Our data indicate that the maturity of the dog, as well as the sex of the dog and sex of the human, influence dog–human interactions. Future studies should examine the specific cues used by dogs to discriminate men and women, perhaps starting with androgen-based olfactory cues, and determine the cause(s) of the change in response to men as the dogs mature.

## Figures and Tables

**Table 1:** Descriptive statistics based on raw data for rate of urination (mean ± *SD*) by male and female shelter dogs (*n* = 113), categorized as either juvenile or mature, during a 20 min walk by either an unfamiliar male or an unfamiliar female. Percentages of walks in which dogs defecated also are shown.

Dog’s Sex	Walker’s Sex	Dog’s Maturity ^1^	Urination Rate ^2^	% Walks with Defecation
Male	Male	Juvenile	0.07 ± 0.03	50.0
Male	Male	Mature	0.18 ± 0.13	41.7
Male	Female	Juvenile	0.10 ± 0.07	71.4
Male	Female	Mature	0.38 ± 0.32	54.2
Female	Male	Juvenile	0.04 ± 0.02	28.6
Female	Male	Mature	0.09 ± 0.07	23.5
Female	Female	Juvenile	0.09 ± 0.05	50.0
Female	Female	Mature	0.12 ± 0.12	68.8

^1^ Juveniles (4 months to <1 year); mature (≥1 year). ^2^ Total number of urinations/20 min.

**Table 2:** Descriptive statistics based on raw data for rate of urination (Mean ± *SD*) by male and female shelter dogs, categorized as either juvenile or mature, during 20 min walks by either a male or a female. Results for the first four walks are included (*n* = 81 dogs for the first two walks; *n* = 68 dogs for walk 3; *n* = 49 dogs for walk 4).

Dog’s Sex	Walker’s Sex	Dog’s Maturity ^1^	Walk 1	Walk 2	Walk 3	Walk 4
Male	Male	Juvenile	0.07 ± 0.04	0.06 ± 0.03	0.07 ± 0.06	0.08 ± 0.08
Male	Male	Mature	0.17 ± 0.11	0.14 ± 0.10	0.16 ± 0.08	0.11 ± 0.05
Male	Female	Juvenile	0.08 ± 0.08	0.09 ± 0.06	0.11 ± 0.08	0.09 ± 0.02
Male	Female	Mature	0.36 ± 0.23	0.39 ± 0.25	0.37 ± 0.22	0.38 ± 0.22
Female	Male	Juvenile	0.05 ± 0.02	0.05 ± 0.03	0.07 ± 0.04	0.03 ± 0.04
Female	Male	Mature	0.08 ± 0.06	0.09 ± 0.06	0.10 ± 0.06	0.09 ± 0.06
Female	Female	Juvenile	0.06 ± 0.04	0.06 ± 0.03	0.05 ± 0.03	0.09 ± 0.06
Female	Female	Mature	0.13 ± 0.10	0.15 ± 0.13	0.16 ± 0.16	0.18 ± 0.15

^1^ Juveniles (4 months to <1 year); mature (≥1 year).

**Table 3:** Percentages of 20 min walks based on raw data in which male and female shelter dogs, categorized as either juvenile or mature, defecated. Results for the first four walks are included (*n* = 81 dogs for the first two walks; *n* = 68 dogs for walk 3; *n* = 49 dogs for walk 4).

Dog’s Sex	Walker’s Sex	Dog’s Maturity ^1^	Walk 1	Walk 2	Walk 3	Walk 4
Male	Male	Juvenile	63.6	44.4	33.3	66.7
Male	Male	Mature	37.5	29.4	37.5	0.00
Male	Female	Juvenile	60.0	64.3	44.4	60.0
Male	Female	Mature	61.4	74.4	69.2	70.0
Female	Male	Juvenile	20.0	40.0	60.0	0.00
Female	Male	Mature	36.6	42.9	36.8	46.7
Female	Female	Juvenile	54.2	60.0	75.0	50.0
Female	Female	Mature	63.2	77.3	68.8	81.8

^1^ Juveniles (4 months to <1 year); mature (≥1 year).

## Data Availability

Data are available online as Supplementary Material.

## Supplementary Materials

The following supporting information can be downloaded at: https://www.mdpi.com/article/10.3390/ani13233649/s1, Table S1: Data on Urination and Defecation by Shelter Dogs.
